# The Importance of Preserving the Teeth and Dental Ethnography

**Published:** 1880-01

**Authors:** 


### BIBLIOGRAPHICAL.

---

*The Importance of Preserving the Teeth, and Dental Ethnography.* By Thomas L. Sydner, D. D. S., of Danville, Va.

This is a small work prepared for the public, in which the author has endeavored to exclude, as far as practicable, technical terms, and render it a practical treatise.

Commencing with the relations of the teeth to the system, their functions and effects upon speech, health, character and features, the author dwells at considerable length upon the most common of their diseases—dental caries, showing the influence of acids, etc., systemic causes, necessity for frequent examinations, and the prevention of decay.

Many subjects relating to the development and formation of the teeth, their eruption, and the care necessary for both temporary and permanent teeth are dwelt upon. Dentonomy or dental physiognomy also claims attention, in which the physiological characteristics of the teeth are described, and the influence of temperament referred to. The author has succeeded in presenting a very readable little work, which must prove serviceable to the public at large, and especially to such patients of the writer as feel interested in the care of their own and of their children's teeth. The work is illustrated with cuts relating to the eruption of the temporary and permanent teeth.

